# Alternative splicing in tea plants was extensively triggered by drought, heat and their combined stresses

**DOI:** 10.7717/peerj.8258

**Published:** 2020-01-29

**Authors:** Yiqian Ding, Yu Wang, Chen Qiu, Wenjun Qian, Hui Xie, Zhaotang Ding

**Affiliations:** Tea Research Institute, Qingdao Agricultural University, Qingdao, China

**Keywords:** *Camellia sinensis* (L.) O. Kuntze, Alternative splicing, Drought stress, Heat stress, RNA-Seq

## Abstract

Drought and heat stresses can influence the expressions of genes, and thereby affect the growth and development of plants. Alternative splicing (AS) of genes plays crucial roles through increasing transcriptome diversity in plant stress responses. Tea plants, widely cultivated in the tropics and subtropics, are often simultaneously exposed to drought and heat stresses. In the present study, we performed a global transcriptome of tea leaves treated with drought, heat or their combination. In total, 19,019, 20,025 and 20,253 genes underwent AS in response to drought (DT), heat (HT) and their combined stress (HD), respectively, of which 12,178, 11,912 and 14,413 genes differentially spliced in response to DT, HT and HD, respectively. Also, 2,447 specific differentially spliced genes (DSGs) were found only in response to HD. All DSGs accounted for  48% of the annotated genes in tea tree genome. Comparison of DSGs and differentially expressive genes (DEGs) showed that the proportions of HT and HD-induced DSGs were 13.4% and 9.2%, while the proportion of DT increased to 28.1%. Moreover, the DEG-DSG overlapped genes tended to be enriched in a wide large of pathways in response to DT. The results indicated that the AS of genes in tea leaves was extensively triggered by drought, heat and their combined stresses. In addition, the AS enhanced the transcriptome adaption in response to drought and heat stresses, and the AS also provoked specific molecular functions in response to drought and heat synergy stress. The study might have practical significance for molecular genetic breeding of tea plants with stress resistance.

## Introduction

Alternative splicing of genes refers to pre-mRNA processing events that multiple mature mRNAs arise from a single gene locus due to alternate splice-site choices. The AS of pre-mRNAs from multiexon genes allows organisms to increase their coding potential and regulate the expressions of genes through multiple mechanisms (Reddy & Barta, 2013). Recent transcriptome-wide analysis of AS revealed that AS was highly pervasive in plants (Syed et al., 2012; Reddy & Barta, 2013). The AS of genes plays crucial roles in plant growth, development and stress responses through affecting transcript abundance, increasing transcriptome diversity and enhancing the functional diversity of proteins (Keller et al., 2016; Thatcher et al., 2016; Jiang et al., 2017). However, to our knowledge, the information regarding the roles of AS in tea plants is still inadequate.

Tea plants, widely cultivated in the tropics and subtropics, are often simultaneously exposed to drought and heat stresses under natural environment, which might cause adverse effects on the growth and development of tea plants. Previous studies showed that regulation of gene transcription played important roles in tea plants responding to drought or heat stress. A few functional genes of tea plants were identified in the responsive process of drought or heat stress, such as Ca^2+^—activated calcium-dependent protein kinase (*CDPK*), nonexpresser of pathogenesis-related genes (*CsNPR1*), and mitogen-activated protein kinase (*MAPK*) (Liu et al., 2016a; Wang et al., 2018). These genes could regulate photosynthesis, energy metabolism, antioxidant metabolism, phytohormone metabolism and signaling pathway of tea leaves. In addition, a number of transcription factors (TFs) in tea plants were identified in response to drought stress or heat stress, such as heat shock transcription factors (*CsHSFs*), *CsWRKY* and homeodomain-leucine zipper (*CsHDZ*). These TFs were involved in the regulation of thermotolerance and drought stress (Wu et al., 2016; Liu et al., 2016b; Shen et al., 2018). As for AS in tea plants, previous studies showed that most genes involved in secondary metabolism occurred AS events, and produced tissue-specific transcripts (Zhu et al., 2018b). The AS of six lipoxygenase genes in tea plants compensated or competed with corresponding full-length transcripts in response to cold, insect attack and phytohormone stresses (Zhu et al., 2018a). However, the comprehensive research about AS in tea plants responding to drought, heat or their combination is still not reported.

In the present study, for further exploring AS events in tea plants responding to some vital abiotic stresses, we performed the global transcriptome of tea leaves treated with drought, heat and their combination using RNA-Seq technology. The DSGs were identified, and their Gene Ontology (GO) and Kyoto Encyclopedia of Genes and Genomes (KEGG) enrichment were analyzed. We further performed the comparision of DSGs and DEGs. The GO and KEGG enrichment of DEG-specific, DSG-specific and DEG-DSG overlapped genes were also analyzed. The results revealed the possible roles of AS in tea leaves in response to drought or heat stress. Therefore, the study might have beneficial to stress-resistance breeding of tea plants from traditional to molecular genetic breeding.

## Material and Methods

### Plant materials for stress treatments and physiological experiments

Two-year-old tea plant, *C. sinensis* (L.) O. Kuntze cv. ‘Zhongcha108’ was used as plant material. Previous studies showed that it has characteristics of high yield and disease-resistance (Wang et al., 2016; Luo et al., 2016), it is widely cultivated in Shandong of China. The tea plants were culture-grown under a 12 h light (25 °C)/12 h dark (20 °C) photoperiod with 1800 Lx photos m^−2^ s^−2^ light intensity and 75% humidity in growth chamber for 2 weeks (Zheng et al., 2016). According to our former study (Zheng et al., 2016), the third, fourth and fifth leaf (function leaves) were chosen as experimental materials for physiological analysis and RNA sequencing. In the study, for drought stress, the tea plants were treated by gradual withholding water for 96 h. For heat stress, the tea plants were subjected to increase temperatures for acclimation (30/25 °C for 48 h, 35/30 °C for 24 h and 40/35 °C for 24 h) at 96 h. For combined HD stress, the tea plants were simultaneously treated by gradual withholding water and increasing temperatures of acclimation for 96 h.

Maximum photochemical quantum yield of PS II (*Fv/Fm*), leaf water content (LWC) and relative electrolyte leakage (REL) of tea leaves at 0, 24, 48, 72 and 96 h were performed for physiological experiments. And the collections were repeated with three times. *Fv/Fm*, LWC and REL were determined as described previously (Su et al., 2015).

### RNA sequencing

For RNA sequencing, the leaf materials were harvested at 96 h. All samples were immediately frozen in liquid nitrogen and stored at −80 °C for transcriptome analysis. Twelve RNA samples (each condition with three biological repeats) were extracted using TRIzol reagent according to the manufactur’s instructions. RNA concentration and integrity were measured using NanoDrop 2000 spectrophotometer and Agilent 2100 Bioanalyzer. Pair-end (PE) sequencing libraries with average insert size of 200 bp were prepared with TruSeq RNA Sample Preparation Kit v2 and performed on HiSeq (Illumina). Error correction was performed using Cutadapt and PerlScript1 (Shanghai Personal Biotechnology Co., Ltd.). Clean reads were obtained after removing the low quality reads and then they were mapped to reference genome (http://www.Plantkingdomgdb.com/tea_tree/) by Tophat2 (with parameters microexon-search and library-type = fr-firststrand). Lastly, RSeQC was used to evaluate the comparison results (Wang, Wang & Li, 2015).

### Detection and identification of alternative splicing events

To identify AS events of tea leaves in responsive to stresses, Stringtie was used to assemble, quantify the transcripts and further detected AS events. ASprofile software (http://ccb.jhu.edu/software/ASprofile/) was used to classify AS events. SpliceGrapher was used to acquire the graph constructed from the gene models, the splice junctions that were recapitulated in the RNA-Seq, and the read depth along the genomic region (Rogers et al., 2012). PerlScript2 (Shanghai Personal Biotechnology Co., Ltd.) was used to filter differential AS events under DT, HT and HD, which splicing positions or types were different from those under CK.

### Analysis of differentially expressed genes and differentially spliced genes

Differential analysis of gene expressions was performed by DESeq (version 1.18.0) programs. Genes with |log_2_fold change| > 1 and *p*-value <0.05 were considered as DEGs. Genes containing differential AS events were considers as DSGs. GO enrichment analysis of DEGs and DSGs were performed by GO Term Finder (https://metacpan.org/release/GO-TermFinder). KEGG analysis of DEGs and DSGs were determined by hypergeometric distribution calculation compared with whole genome background. The significantly enriched GO terms and KEGG pathways were selected based upon a *p*-value cut-off of 0.05.

### Validation of AS events by reverse transcription-polymerase chain reaction (RT-PCR)

Total RNA was treated with DNase I and reverse-transcribed using oligo-dT primers (PrimerScript II RTase, TaKaRa) according to the manufacturer’s instructions. RT-PCR was performed in a 25 µl reaction volume using primers flanking the AS site. The primers used for RT-PCR analysis were showed in Table S2.

### Validation of AS gene expressions by quantitative real-time polymerase chain reation (qRT-PCR)

To verify RNA-seq results, 8 AS genes were selected for qRT-PCR test. QRT-PCR was performed using SYBR Green PCR Master Mix (Takara) and run on LightCycler 480 Real-Time PCR System (Roche Applied Science) under the following parameters: 95 °C for 30 s, 40 cycles at 95 °C for 5 s, 60 °C for 30 s. Triplicates of each reaction were performed, and glyceraldehyde-3-phosphate dehydrogenase (GAPDH) sequence was used as internal reference control. Relative expression levels were calculated using the 2^−ΔΔ*Ct*^ method and normalized to the actin gene (Livak, 2001). The details of the primers used in qRT-PCR were given in Table S1.

### Analysis of gene structures and amino acid sequences

Gene structures were analyzed with online website Gene Structure Display Server (GSDS 2.0, http://gsds.cbi.pku.edu.cn/index.php). The amino acid sequences were analyzed with online website Open Reading Frame Finder (ORFs, https://www.ncbi.nlm.nih.gov/orffinder/).

## Results

### Physiological characterization of tea plants under DT, HT and HD

**Figure 1:**
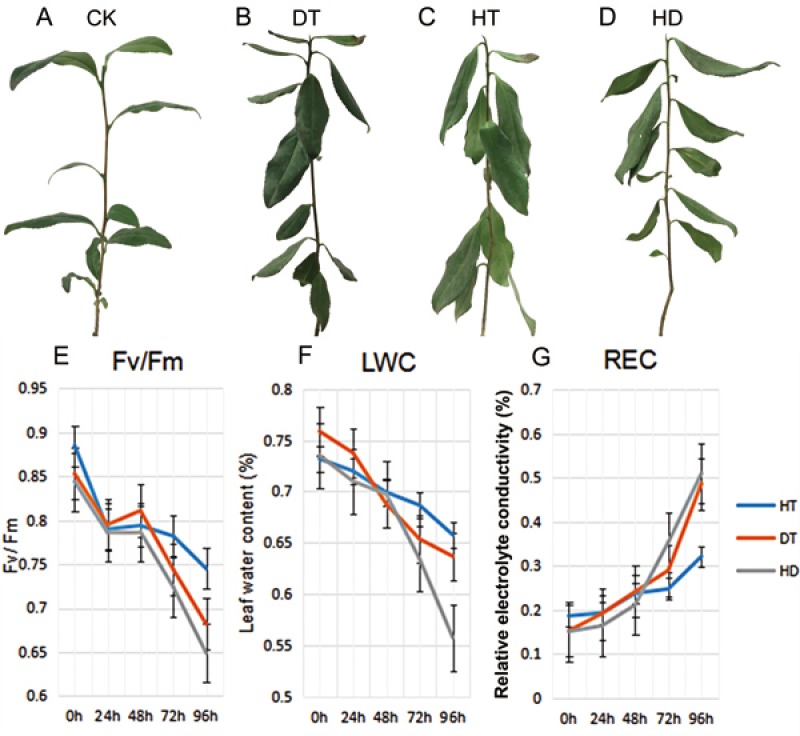
The phenotypes and physiological analyses of tea plants under CK, DT, HT and HD. (A) A good tea plant was found after 96 h of normal condition. (B) A number of wilted and few curly leaves were found after 96 h of drought treatment (DT). (C) A few mild wilting leaves were found after 96 h of heat treatment (HT). (D) Many distinct wrinkles and brittle leaves were found after 96 h of combined drought and heat treatment (HD). (E) Leaf maximum photochemical quantum yield of PS II (*Fv*/*Fm*) values were determined at five time-points (0, 24, 48, 72 and 96 h) during DT, HT and HD. (F) Leaf water content (LWC) values were determined at five time-points during DT, HT and HD. (G) Leaf electrolyte conductivity (REC) values were determined at five time-points during DT, HT and HD. DT, drought stress; HT, heat stress; HD, heat and drought stress.

To validate the injury of tea leaves exposed to different stresses, we observed the phenotypic changes and performed physiological index of tea leaves treated with DT, HT and HD. A number of wilted and few curly leaves were found under DT, a few mild wilting leaves were found under HT. While a lot of distinct wrinkle and brittle leaves were found under HD (Figs. 1A–1D). Accordingly, we examined the *Fv/Fm*, LWC and REC of tea leaves at five time-points (0, 24, 48, 72 and 96 h). The values of *Fv/Fm* and LWC reduced significantly, while REC increased significantly under DT and HT, which were emphasized even more under HD (Figs. 1E–1G). The results indicated that both heat and drought caused dehydration in tea leaves, which caused damage to photosynthesis system and membrane.

### Analysis of biological functions in DEGs responding to DT, HT and HD

To understand how genes were regulated in tea leaves under stresses, we identified DEGs and performed the enrichment analysis of GO terms, KEGG pathways in tea leaves under DT, HT and HD. In total, 10,136, 4,768 and 2,838 DEGs were identified in response to DT, HT and HD. Among the DEGs, 4,649, 2,470 and 1,725 were up-regulated in response to DT, HT and HD, 5,487, 2,298 and 1,113 were down-regulated in response to DT, HT and HD, respectively (Fig. S1). After performed GO enrichment analysis, 49, five and six GO terms exhibited significant enrichment in DEGs in response to DT, HT and HD, respectively (Fig. S2A). There was little overlap among the significantly enriched GO terms of DEGs in response to DT, HT and HD. For example, cellular amino acid metabolic process, zinc ion binding and oxidation–reduction process showed significant enrichment in response to DT, HT and HD, respectively (Fig. S2B). After performed KEGG enrichment analysis, 46, 35 and 26 KEGG pathways exhibited significant enrichment in DEGs in response to DT, HT and HD, respectively (Fig. S3A). There was some overlap among the significantly enriched KEGG pathways of DEGs in response to DT, HT and HD. For example, photosynthesis and galactose metabolism pathways both exhibited significant enrichment in response to DT, HT and HD (Fig. S3B). Apart from the shared stress responses, the three stresses triggered different responses. For example, carbon fixation in photosynthetic organisms was strong triggered by DT, steroid biosynthesis was triggered by HT, while phenylpropanoid biosynthesis in DEGs was weakly triggered by HD.

### Analysis of AS events responding to DT, HT and HD

**Figure 2:**
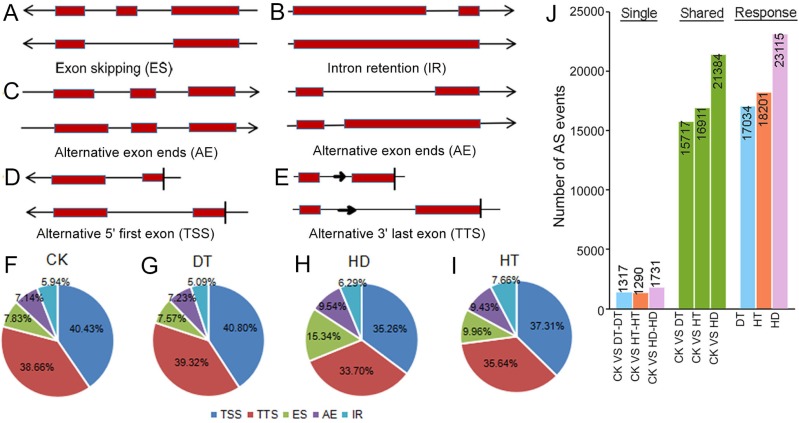
Summary of alternative splicing (AS) events in tea leaves. (A) Schematic representation of intron retention (IR), (B) exon skipping (ES), (C) alternative exon ends (AE), (D) alternative 5′ first exon (TSS) and (E) alternative 3′ last exon (TTS). (F) Statistics of the number of five AS events under CK, (G) DT, (H) HT and (I) HD. (J) The histogram showing the number of common and specific AS events during DT, HT and HD. The histogram of CK VS DT-DT: the number of AS events only detected in DT compared with CK. The histogram of CK VS DT: the number of common AS events detected in CK and DT. The histogram of DT: total number of AS events detected in DT.

To examine changes on AS in response to stresses, we performed comprehensive profilings of AS landscapes in tea leaves under DT, HT and HD. Totally, 66475, 59835, 76682 and 72120 AS events were identified in response to CK, DT, HT and HD, corresponding to 20041, 19019, 20025 and 20253 genes, respectively. On the average, each AS gene contained three to four AS events under all conditions. All AS events were distributed in 23771 genes, which accounted for ∼64% of the annotated genes in tea tree genome (36591). Among the annotated genes, 31165 had two or more exons (referred to as multiexonic genes). In this aspect, 70.5% of the multiexonic genes were alternatively spliced in tea leaves. Moreover, all AS events were divided into five types, including intron retention (IR), exon skipping (ES), alternative exon ends (AE), alternative 5′ first exon (TSS) and alternative 3′ last exon (TTS) (Figs. 2A–2E). TSS represented 35.26∼40.80% (40.43%, 40.80%, 37.31% and 35.26% under CK, DT, HT and HD, respectively) of the total AS events and was the most abundant type, followed by TTS (38.66%, 39.32%, 35.64% and 33.70%), ES (7.83%, 7.57%, 9.96% and 15.34%), AE (7.14%, 7.23%, 9.43% and 9.54% and) and IR (5.94%, 5.09%, 7.66% and 6.29%) (Figs. 2F–2I). The results showed that the ratios of five AS types were roughly the same when compared within different conditions. After comparing AS models under three stresses with normal conditions, 17034, 18201, 23115 differential AS events were found in responsive to DT, HT or HD, respectively (Fig. 2J), indicating that AS events in tea leaves were widely induced by DT, HT and HD, especially induced by HD.

**Figure 3:**
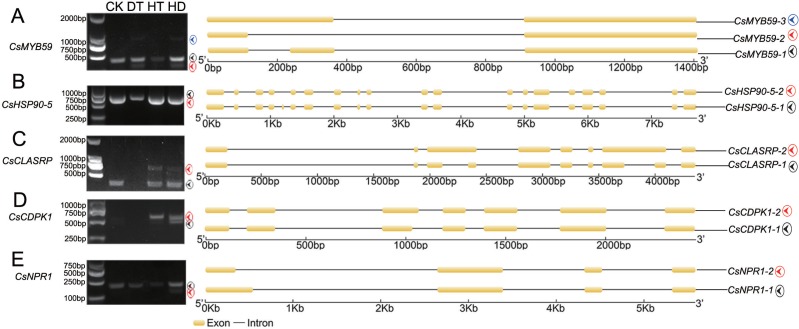
Experimental validation of stress responsive AS events by reverse transcription RT-PCR in tea leaves. Five AS genes (under CK, DT, HT and HD) were analyzed using RT-PCR to validate the RNA-Seq results. The RT-PCR validation confirmed the computational prediction of AS genes including (A) *CsMYB59*, (B) *CsHSP90-5* (heat shock protein 90-5), (C) *CsCLASRP* (CLK4-associating serine/arginine rich protein), (D) *CsCDPK1* (Calcium-dependent protein kinases) and (E) *CsNPR1* (nonexpressor of pathogenesis-related genes).

To test the accuracy of our RNA- Seq data, qRT-PCR was performed on 8 AS genes, and we found the data from both were highly consistent (Fig. S4). RT-PCR was also performed on 5 AS genes to verify the stress responsive AS events (Fig. 3). *CsMYB59* detected three transcripts, CLK4-associating serine/arginine rich protein (*CsCLASRP*), heat shock protein 90-5 (*CsHSP90-5*), *CsNPR1* and *CsCDPK1* detected two transcripts.

### Analysis of biological functions in DSGs responding to DT, HT and HD

**Figure 4:**
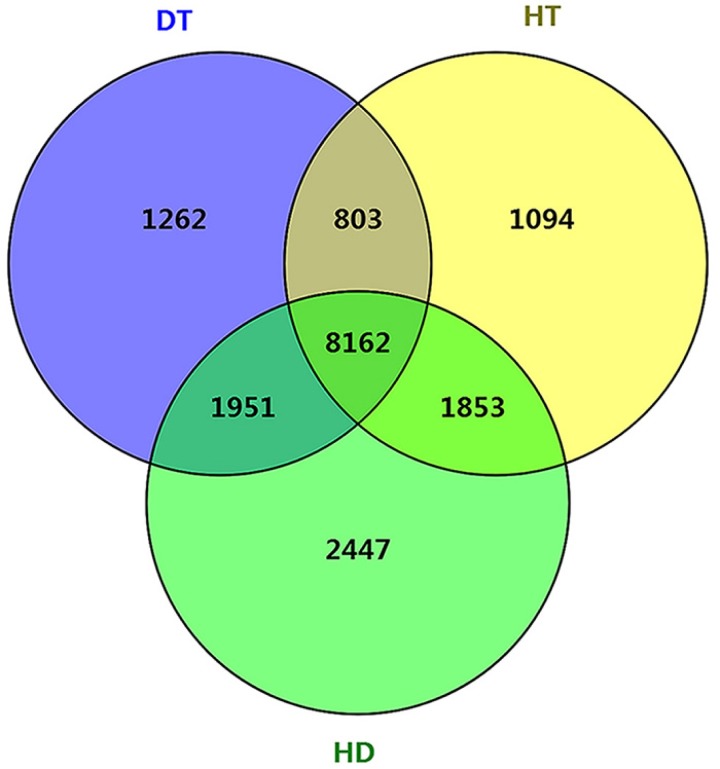
Comparison of the number of differentially spliced genes (DSGs) in response to DT, HT and HD in tea leaves. Venn diagram showing that the overlap of DSGs among DT, HT and HD. Genes containing differential AS events were considers as DSGs.

To further examine the molecular differences between single and combined stress responses, we identified DSGs in response to DT, HT and HD. In total, 12178, 11912 and 14413 DSGs were identified in response to DT, HT and HD compared with CK. Among the DSGs, 2072, 1082 and 927 were up-regulated (Fig. S5), respectively. Four types of key transcription factors exhibited significant AS pattern changes to respond to DT, HT or HD, including 10 NAC (e.g., *CSA031084*, *CSA004675*), 33 WRKY (e.g., *CSA021419*, *CSA028948*), 15 bZIP (e.g., *CSA003423*, *CSA025946*) and 55 MYB (e.g.*CSA014712*, *CSA007325*) transcription factors (Table S3). In addition, 14, 15 and 20 HSPs, such as *CsHSP90-5* and *CsHSP40*, were found to be differential spliced under DT, HT or HD (Table S4). Moreover, a large number of DSGs (8162, 46.4% of all DEGs) were found to be modulated by three stress conditions (Fig. 4). The overlap DSGs of DT/HD were no significant difference from that of HT/HD, with the numbers of 10113 compared to 10015. In addition, 2447 DSGs (e.g., *CsCDPK1*, *CsNPR1*, *CsHSFA9* and *CsIAA27* ) were uniquely responsive to HD, which were significantly higher than that in response to DT and HT (1262 and 1094). The results showed that the numbers of DSGs in HD were more than that in DT and HT.

**Figure 5:**
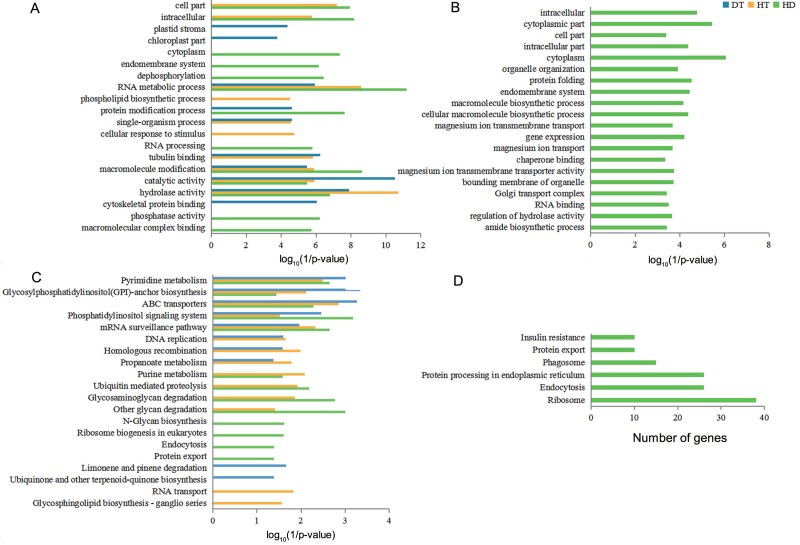
GO terms and KEGG pathways analysis of DSGs under DT, HT and HD. (A) The bar chart showing 20 GO terms that significantly enriched in DSGs in response to DT, HT and HD. (B) The bar chart showing 20 GO terms that significantly enriched in HD-specific DSGs. (C) The bar chart showing 20 KEGG pathways that significantly enriched in DSGs in response to DT, HT and HD. (D) The bar chart showing all KEGG pathways that significantly enriched in HD-specific DSGs. The significantly enriched GO terms and KEGG pathways were selected based upon a *p*-value cut-off of 0.05.

To understand the functional differences of DSGs in tea leaves under DT, HT and HD, we performed the enrichment analysis of GO and KEGG pathways to explore the biological functions. The significantly enriched GO terms of DT, HT, and HD were similar and all included terms associated with RNA metabolic process, macromolecule modification, catalytic activity and hydrolase activity (Fig. 5A). In spite of the common modulations, the three stresses triggered different responses. For example, the GO terms related to cytoskeletal protein binding was triggered by DT, phospholipid biosynthetic process was triggered by HT, while phosphatase activity in DSGs was triggered by HD. In addition, HD-specific DSGs were mainly enriched in protein folding, gene expression, organelle organization, magnesium ion transmembrane transporter activity and chaperone binding (Fig. 5B). While the DSGs of DT and HT were less enriched in these GO terms. The significantly enriched KEGG pathways of DT, HT and HD were similar and all included mRNA surveillance pathway, pyrimidine metabolism, glycosylphosphatidyli-nositol(GPI) - anchor biosynthesis, ABC transporters and phosphatidylinositol signaling system (Fig. 5C). Apart from these shared stress responses, the combined stress invoked different pathways. For example, HD-specific DSGs were mainly enriched in endocytosis, phagosome, protein export, insulin resistance, ribosome and protein processing in endoplasmic reticulum (Fig. 5D). While the DSGs of DT and HT were less enriched in these pathways. Taken together, these results showed that HD could induce different responses in tea leaves compared with DT and HT.

### Comparative analysis of DSGs and DEGs

**Figure 6:**
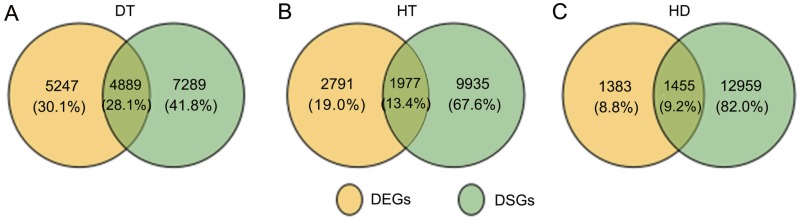
Comparison of the proportion of differentially expressed (DEGs) and DSGs in response to DT, HT and HD in tea leaves. (A) Venn diagram showing that the overlap of DEGs and DSG sunder DT, (B) HT and (C) HD. Genes with |log_2_fold change| > 1 and *p*-value < 0.05 were considered as DEGs.

To further clarify the relationship between AS and transcription, we conducted the comparition of the genes undergoing AS and transcription changes in response to DT, HT and HD. By compared, there were 1977 DSGs (13.4% of all HT-DSGs) differentially expressed in response to HT, and 1455 DSGs (9.2% of all HD-DSGs) also differentially expressed in response to HD (Fig. 6). While under DT, the numbers of DSGs which were also identified as DEGs increased to 4889 (28.1% of all DT-DSGs). The results showed that there were more overlaps under DT, whereas there were a little overlaps between DSGs and DEGs in HT and HD responses. In addition, there were 7289, 9935 and 12959 DSG-specific genes in response to DT, HT and HD, respectively. The results indicated that these genes were mainly subjected to AS modulation in response to DT, HT and HD.

**Figure 7:**
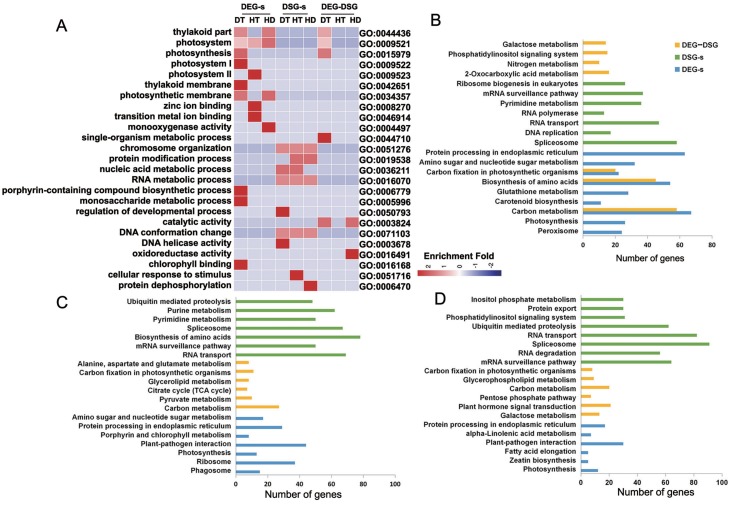
GO terms and KEGG pathways analysis of DEG-specific, DSG-specific and DEG-DSG overlapped genes in response to DT, HT and HD. (A) The heatmap showing the enriched GO terms of DEG-specific, DSG-specific and DEG-DSG overlapped genes. DEGs, DEG-specific genes; HD-s, HD-specific genes; DSGs, DSG-specific genes; DEG-DSG, DEG and DSG overlapped genes. The color scale represents enrichment folds after *Z*-score of different GO terms. (B–D) The bar diagrams showing the enriched KEGG pathways of DEG-specific, DSG-specific and DEG-DSG overlapped genes.

To research the biological functions regulated by AS and transcription, we performed the enrichment analysis of GO terms on DEG-specific, DSG-specific and DEG-DSG overlapped genes. Numbers of significantly (corrected *p*-value ≤ 0.05) enriched GO terms for DEG-specific genes were 25 (DT), 8 (HT) and 9 (HD) (Table S5). There were 26 (DT), 41 (HT) and 68 (HD) GO terms significantly enriched for DSG-specific genes. There were 16 (DT), 0 (HT) and 2 (HD) GO terms significantly enriched for DEG-DSG overlapped genes. Under DT, DEG-specific genes were mainly enriched in photosynthetic membrane, porphyrin-containing compound biosynthetic process and monosaccharide metabolic process (Fig. 7A). DSG-specific genes were largely enriched in chromosome organization, DNA conformation change and RNA metabolic process. As for DEG-DSG overlapped genes under DT, they were only enriched in photosynthesis, including plastid part, chloroplast part and thylakoid part. Under HT, DEG-specific genes were mainly enriched in zinc ion binding, transition metal ion binding and photosystem. DSG-specific genes were largely enriched in protein modification process, nucleic acid metabolic process, RNA metabolic process and cellular response to stimulus. As for HD, DEG-specific genes were enriched in photosynthetic membrane and monooxygenase activity, etc. DSG-specific genes were enriched in protein dephosphorylation and protein modification process, etc. Whereas DEG-DSG overlapped genes under HD were only enriched in catalytic activity and oxidoreductase activity.

We also performed the enrichment analysis of KEGG pathways on DEG-specific, DSG-specific and DEG-DSG overlapped genes to research the relevant pathways regulated by AS and transcription. Numbers of significantly (*p*-value ≤ 0.05) enriched pathways for DEG-specific genes were 22 (DT), 7 (HT) and 8 (HD) (Table S6). There were 12 (DT), 24 (HT) and 22 (HD) pathways significantly enriched for DSG-specific genes.There were 20 (DT), 10 (HT) and 8 (HD) pathways significantly enriched for DEG-DSG overlapped genes. Under DT, there were 67, 63 and 54 DEG-specific genes enriched in carbon metabolism, protein processing in endoplasmic reticulum and biosynthesis of amino acids, respectively. There were 58, 48 and 37 DSG-specific genes enriched in spliceosome, RNA transport and mRNA surveillance pathway. There were 58, 45 and 24 DEG-DSG overlapped genes enriched in carbon metabolism, biosynthesis of amino acids and Glycolysis/Gluconeogenesis (Fig. 7B). Under HT, there were 44, 29 and 37 DEG-specific genes enriched in plant-pathogen interaction, protein processing in endoplasmic reticulum and ribosome, respectively. There were 78, 62 and 69 DSG-specific genes enriched in biosynthesis of amino acids, purine metabolism and RNA transport. There were 27, 11 and 10 DEG-DSG overlapped genes enriched in carbon metabolism, carbon fixation in photosynthetic organisms and pyruvate metabolism (Fig. 7C). As for HD, there were 30,12 and 5 DEG-specific genes enriched in plant-pathogen interaction, photosynthesis and zeatin biosynthesis pathways, respectively. There were 82, 62 and 31 DSG-specific genes enriched in RNA transport, ubiquitin mediated proteolysis and phosphatidylinositol signaling system. There were 21, 9 and 20 DEG-DSG overlapped genes enriched in plant hormone signal transduction, glycerophospholipid metabolism and carbon metabolism pathways (Fig. 7D).

## Discussion

### The AS enhanced the transcriptome adaption in response to drought and heat stresses

Plants are subjected to various environmental stresses during their growth and development processes, and to cope with these stresses they have provoked AS at post-transcriptional levels (Yanting et al., 2014; Dorothee & Brown, 2013). Up to date, the biological functions of AS in plants were defined in many genes, especially those involved in the regulation of stress responses, such as TFs. For example, in rice, *HSFA2* and *DREB2*, two key abiotic stress regulators, occurred AS in response to drought and heat stresses. The transcripts of *HSFA2* and *DREB2* encoded proteins with the activity of transcriptional activation, which dramatically promoted the expressions of responsive genes and enhanced the drought and heat tolerance (Matsukura et al., 2010; Cheng et al., 2015). However, in tea plants, the report of AS genes in response to drought and heat stresses is still absent.

In the present study, 12178, 11912 and 14413 genes underwent differential AS in response to DT, HT and HD, respectively. There were 13, 15 and 14 ser/arg-rich SR genes in tea leaves were found to be differentially spliced in response to DT, HT and HD (Table S7). Previous study showed that SR proteins participated in the process of RNA-protein and protein-protein interactions during spliceosome assembly (Tacke & Manley, 1999). They were demonstrated to be key regulators of plant responses to environmental conditions (Saiprasad Goud, Gul Shad & Reddy; Filichkin et al., 2015). It has been shown that the AS of *Arabidopsis* SR genes was altered by various stresses, raising the possibility of rapid reprogramming of the whole transcriptome by external signals (Reddy & Shad, 2011). In the study, *CsCLASRP*, a splicing factor, was not differentially expressed in response to DT, HT and HD, but underwent differential AS with IR event in response to HT and HD, suggesting that the AS of *CsCLASRP* might regulate the abundance of full-length transcripts via compensation of splicing machinery. The AS transcript of *CsCLASRP* introduced premature termination codons, and translated into truncated protein. We speculated that the truncated protein of *CsCLASRP* might have different functions compared with the protein translated from full-length transcript. And the AS of *CsCLASRP* in response to heat stress could regulate the AS of other stress-responsive genes to enhance the transcriptome adaption. But the regulations of AS on SR genes in tea plants responding to abiotic stress need to be further investigated.

**Figure 8:**
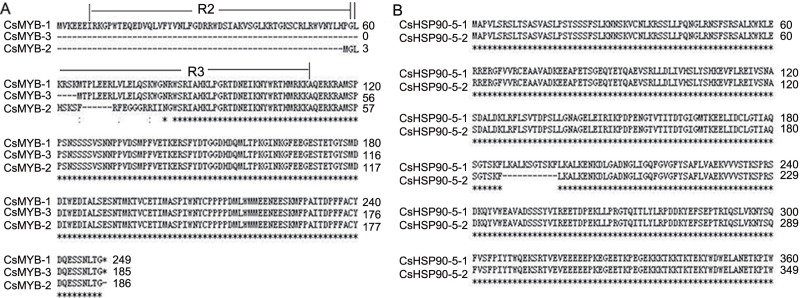
The amino acids sequences analysis of two AS genes. (A) The amino acids sequences analysis of *CsMYB59*. The amino acids sequences of CsMYB59-1, CsMYB59-2 and CsMYB59-3 differed only in their MYB repeats. (B) The amino acids sequences analysis of *CsHSP90-5*. The amino acids sequence of CsHSP90-5-2 missed 11 continuous amino acids compared with that of CsHSP90-5-1.

Notably, some important genes modulated by AS in tea plants were also found in other plants, such as *AtMYB59* and *AtHSP90-5* (Fasani et al., 2019; Li et al., 2006; Cao et al., 2010; Oh et al., 2014). Previous studies showed that MYB transcription factor genes played central roles in various defence responses of plants (Li et al., 2006). In *Arabidopsis*, *AtMYB59* was involved in stress responses as a negative regulator of calcium signalling and homeostasis (Fasani et al., 2019). Here, in tea plants, *CsMYB59* was up-regulated in response to DT, but had no significant change in response to HD. However, *CsMYB59* occurred differential AS with IR and ES events in response to DT and HD, suggesting that the AS of *CsMYB59* might regulate the abundance of full-length transcripts via synergy or competition of splicing machinery. Moreover, *CsMYB59* was involved in the pathway of plant-pathogen interaction, it seemed probably that the AS of *CsMYB59* might promote this pathway in response to DT, but inhibit this pathway in response to HD. In addition, the transcripts of *CsMYB59* were different from the transcripts of *AtMYB59*, and the analysis of amino acid sequences showed that the alternative transcripts of *CsMYB59* differed only in their MYB repeats(Fig. 8A). Prior study showed that the proteins encoded by transcripts of *AtMYB59* differed only in their MYB repeats, and they might have binding affinities to different target genes (Li et al., 2006). We speculated that *CsMYB59* in tea leaves could encode alternative proteins, which might enhance the transcriptome adaption by binding different target genes.

As for HSP90, it is a chaperone protein that assists other proteins to fold properly, stabilizes proteins against heat stress, and aids in protein degradation. It has been reported that *AtHSP90-5* was a molecular chaperone required for chloroplast biogenesis, and it involved in biological process responding to heat, salt and water deprivation stresses (Cao et al., 2010; Oh et al., 2014; Song et al., 2009). Here, *CsHSP90-5* produced one AS transcript with ES event in response to DT, HT and HD, and the alternative transcript skipped exon 16 of 32 bp size. Further analysis of amino acid sequences showed that CsHSP90-5-2 missed 11 continuous amino acids (LKALKSGTSKF) (Fig. 8B). And *CsHSP90-5* was annotated in unfolded protein binding and protein folding. It seemed probably that the AS of *CsHSP90-5* might modulate the processes of unfold protein response, endoplasmic reticulum stress and the formation of protein structure in response to DT, HT and HD. Taken together, we speculated that the AS of *CsHSP90-5* could enhance the transcriptome adaption in response to heat and drought stresses.

AS might provide a novel strategy of regulating the environmental fitness of tea plants. As candidate AS genes, *CsCLASRP*, *CsMYB59* and *CsHSP90-5* could be used as molecular markers for breeding tea varieties with higher drought resistance.

In summary, up to date, this is the first time to be found that *CsCLASRP*, *CsMYB59* and *CsHSP90-5* occurred AS events in tea plants in response to drought and heat stresses. So, it needs to further study on the functions of AS in these genes.

### The AS and transcription fine-tuned the response of drought stress

The relationship between AS and transcription reflected the complex mechanism of plants in response to abiotic stresses. Previous studies showed that there was almost no overlap between DSGs and DEGs in *Physcomitrella patens* and *Arabidopsis*, and the AS and transcription could function independently in response to heat and salt stresses (Cheng et al., 2015; Ding et al., 2014). However, the relationship between AS and transcription in tea plants treated with abiotic stress is still not reported.

In this study, although the enrichment analysis of KEGG pathways indicated that the overlapped genes under HT or HD were regulated by both AS and transcription, only a small subset of DSGs overlapped with DEGs (9.2% in HT and 13.4% in HD, respectively). Therefore, we suggested that AS and transcription in tea leaves could be separately functioned in response to HT or HD. On the contrary, up to 28.1% DSGs were overlapped with DEGs under DT. The amount was more than that in *Arabidopsis* under salt stress (only 4% of DSGs, Ding et al., 2014). Conformably, the various GO terms and metabolic pathways were significantly over-represented among DEG-DSG overlapped genes in response to DT, including thylakoid part, photosystem I, photosynthesis terms and 2-oxocarboxylic acid metabolism, phosphatidylinositol signaling system and galactose metabolism pathways. The results showed that AS and transcription played important roles in these metabolic pathways in response to drought stress. Therefore, the results suggested that the regulations of AS and transcription could function concertedly in tea leaves to better cope with drought stress.

### The AS provoked specific molecular functions in response to drought and heat synergy stress

Plants are often subjected to the combined heat and drought stresses, especially in the filed conditions. Prior study in wheat indicated that combined heat and drought stresses provoked unique AS responses that can not be induced by individual stress alone (Liu et al., 2018). Former study found that 963 AS events significantly responded to the drought and heat combined stress, but barely changed under individual drought or heat stress, suggesting that these HD-specific AS responses might be caused by the interaction of drought and heat responses. Moreover, some GO terms, such as glutathione biosynthetic process and DNA methylation, significantly enriched for HD-specific AS genes, indicating that these processes tended to be responsive to the combination of drought and heat stress at AS level (Liu et al., 2018). However, there is no report about AS events of tea plants in response to drought and heat synergy stress.

In our study, 2447 AS genes differentially spliced in response to HD but not respond to DT or HT, suggesting that the HD-specific AS responses were only induced by the interaction of drought and heat stresses. Moreover, HD-specific DSGs were mostly enriched in protein export, ribosome and protein processing in endoplasmic reticulum, while DSGs of DT and HT were less enriched in these pathways. Interestingly, several stress responsive genes exhibited HD-specific AS responses were also reported in other plants, such as *CDPKs* and *NPR1* in *Arabidopsis* and rice. *CDPKs* could perceive intracellular changes in Ca^2+^ concentration and translate them into specific phosphorylation events to initiate further downstream signaling processes (Schulz, Herde & Romeis, 2013). In *Arabidopsis*, *CDPKs* phosphorylated the binding motif of Vacuolar K^+^ Channel TPK1 in response to salt stress (Latz et al., 2013). In rice, *OsCPK13*, *OsCPK17*, *OsCPK18* and *OsCPK19* produced AS transcripts, which encode truncated proteins lacking whole or partial domains with different biological functions (Almadanim, Gonçalves & Rosa, 2018). Here, in tea plants, *CsCDPK1* was down-regulated in response to HT, but had no significant change in response to HD. And *CsCDPK1* only occurred AS with AE events in response to HD, suggesting that the AS of *CsCDPK1* might repress the down-regulation of *CsCDPK1* in response to HD. Furthermore, *CsCDPK1* was annotated in protein phosphorylation and protein serine/threonine kinase activity, it seemed probably that the cellular protein modification process might be regulated by the AS of *CsCDPK1*.

As for NPR, it was well known that played an important role in plant disease resistance. In addition, there is some evidence showed that it plays a role in oxidative stress and heat stress. In tobacco, the heterologous expression of *AtNPR1* enhanced the oxidative stress tolerance, which was associated with the constitutive up-regulation of several antioxidants (PR1, PR2, PR5, APX and Cu^2+^/Zn^2+^ SOD) (Srinivasan et al., 2009). In *Arabidopsis*, *NPR1* mutant, which is defective in SA-induced defense responses, showed defective in basal thermotolerance (Jane et al., 2005). Here, in tea plants, *CsNPR1* was up-regulated in response to DT, but had no significant change in response to HD. However, *CsNPR1* also only occurred AS with AE event in response to HD, suggesting that the AS of *CsNPR1* might regulate the abundance of corresponding full-length transcripts via competition of splicing machinery. Moreover, *CsNPR1* was involved in plant hormone signal transduction pathway, it seemed probably that the plant hormone signal transduction pathway might be regulated by the AS of *CsNPR1*. So, we speculated that tea plants require specific responses at both transcriptional and AS levels to adapt to heat and drought synergy stress which usually occurred in the field.

## Conclusions

Through analyzing global changes in AS genes in response to drought, heat or their combined stresses, ∼48% of the annotated genes in tea tree genome were differential spliced. The results showed that AS in tea plants could enhance the transcriptome adaption in response to drought and heat stresses. And the AS and transcription could function concertedly in response to drought stress. Meanwhile, the AS in tea plants under drought and heat synergy stress provoked specific molecular functions, such as protein export and protein processing in endoplasmic reticulum. The study provided the insights into the AS events underlying abiotic stresses and highlighted the potential regulatory functions of AS on stress-response genes of tea plants.

##  Supplemental Information

10.7717/peerj.8258/supp-1 Figure S1 Number of differentially expressive genes identified under DT, HT and HD Genes with |log_2_fold change| > 1 and *p*-value < 0.05 were considered as DEGs.

10.7717/peerj.8258/supp-2 Figure S2 GO enrichment analysis of DEGs under DT, HT and HD (A) The venn diagram showing the number of GO terms significantly enriched in DEGs under DT, HT and HD. (B) The bar chart showing the GO terms that only enriched in HD-specific DEGs.

10.7717/peerj.8258/supp-3 Figure S3 KEGG enrichment analysis of DEGs under DT, HT and HD (A) The venn diagram showing the number of KEGG pathways significantly enriched in DEGs under DT, HT and HD. (B) The bar chart showing the KEGG pathways that only enriched in HD-specific DEGs.

10.7717/peerj.8258/supp-4 Figure S4 QRT-PCR analysis of alternative splicing genes under CK, DT, HT and HD The expression at CK was set as 1, and the relative expression level was calculated for several genes. Bar shows the means ± SD (*n* = 3) of three biological replicates. *CsCAMT3* (Calmodulin-binding transcription activator 3), *CsGOLS* (Galactinol synthase).

10.7717/peerj.8258/supp-5 Figure S5 Number of differentially alternative splicing genes identified under DT, HT and HD

10.7717/peerj.8258/supp-6 Table S1 Primers used in qRT-PCR validation of AS gene expression

10.7717/peerj.8258/supp-7 Table S2 Primers used in RT-PCR validation of stress responsive AS events

10.7717/peerj.8258/supp-8 Table S3 Statistical summary of transcription factors that were alternatively spliced (under DT, HT and HD)

10.7717/peerj.8258/supp-9 Table S4 Statistical summary of HSPs that were alternatively spliced (under DT, HT and HD)

10.7717/peerj.8258/supp-10 Table S5 Statistical summary of GO terms that enriched in DEG-specific, DSG-specific and DEG-DSG overlapped genes (under DT, HT and HD)

10.7717/peerj.8258/supp-11 Table S6 Statistical summary of KEGG pathways that enriched in DEG-specific, DSG-specific and DEG-DSG overlapped genes (under DT, HT and HD)

10.7717/peerj.8258/supp-12 Table S7 Statistical summary of SR genes that were alterantively spliced (under DT, HT and HD)

10.7717/peerj.8258/supp-13 Supplemental Information 13 The raw data for Fig. 1

10.7717/peerj.8258/supp-14 Supplemental Information 14 Complete gel map of six alternative splicing genes including control gene *GAPDH*

10.7717/peerj.8258/supp-15 Supplemental Information 15 The results of data quality control about RNA-Seq

## Additional Information and Declarations

### Data Availability

The following information was supplied regarding data availability: The sequences are available at NCBI SRA: PRJNA545401.
https://www.ncbi.nlm.nih.gov/sra/PRJNA545401.
